# Defining and Predicting HIV Immunological Non-Response: A Multi-Definition Analysis from an Indonesian Cohort

**DOI:** 10.3390/v17121581

**Published:** 2025-12-04

**Authors:** Brian Eka Rachman, Yehuda Tri Nugroho Supranoto, Soraya Isfandiary Iskandar, Tri Pudy Asmarawati, Siti Qamariyah Khairunisa, Muhammad Vitanata Arfijanto, Usman Hadi, Muhammad Miftahussurur, Nasronudin Nasronudin, Masanori Kameoka, Retno Pudji Rahayu, Afif Nurul Hidayati

**Affiliations:** 1 Doctoral Program of Medical Science, Faculty of Medicine, Universitas Airlangga, Surabaya 60131, Indonesia; brian.eka@fk.unair.ac.id; 2 Institute of Tropical Disease, Universitas Airlangga, Surabaya 60115, Indonesia; skhairunisa@staf.unair.ac.id (S.Q.K.);; 3 Department of Internal Medicine, Faculty of Medicine, Universitas Airlangga, Surabaya 60131, Indonesia; 4 Harvard Medical School, Boston, MA 02115, USA; yehuda.supranoto@gmail.com; 5 Department of Public Health and Preventive Medicine, Faculty of Medicine, Universitas Airlangga, Surabaya 60131, Indonesia; 6 Universitas Airlangga Hospital, Surabaya 60116, Indonesia; 7 Helicobacter pylori and Microbiota Study Group, Institute of Tropical Medicine, Universitas Airlangga, Surabaya 60115, Indonesia; 8 Center for Infectious Diseases, Graduate School of Medicine, Kobe University, Kobe 650-0017, Japan; mkameoka@port.kobe-u.ac.jp; 9 Department of Public Health, Graduate School of Health Sciences, Kobe University, Kobe 654-0142, Japan; 10 Department of Oral and Maxillofacial Pathology, Faculty of Dentistry, Universitas Airlangga, Surabaya 60132, Indonesia; 11 Department of Dermatology Venereology and Aesthetics, Faculty of Medicine, Universitas Airlangga, Surabaya 60131, Indonesia

**Keywords:** HIV infections, immune reconstitution, anti-retroviral agents, risk factors, immunological nonresponse

## Abstract

Immunological non-response (INR) to antiretroviral therapy (ART) is a critical concern for PLHIV, characterized by inadequate CD4^+^ T-cell recovery despite virological suppression. This retrospective study analyzed medical records of virologically suppressed adult PLHIV on ART (2004–2024) at two hospitals in Surabaya, Indonesia, using four operational categories to identify clinical and demographic determinants of INR. Patients were classified as immunological responders (IRs) or non-responders (INRs) based on four definitions: INR1 (CD4^+^ gain < 100 cells/mm^3^), INR2 (CD4^+^ < 350 cells/mm^3^), INR3 (meeting of either criterion), and INR4 (meeting of both criteria). Of 464 patients, 382 were analyzed. Baseline CD4^+^ < 200 cells/mm^3^ strongly predicted INR2 (aOR = 5.60, 95% CI: 2.95–10.62) and INR3 (aOR = 4.46, 95% CI: 2.39–8.29), while anal sexual transmission was protective against INR2 (aOR = 0.42, 95% CI: 0.19–0.92) and INR3 (aOR = 0.41, 95% CI: 0.19–0.89). By month 12, IR groups had over 350 CD4^+^ cells/mm^3^, with faster recovery slopes in months 0–6 (IR: >20 vs. INR: <10 cells/mm^3^/month). INR1 and INR4 had flat or negative slopes at 12–24 months, while IR groups had positive slopes. Baseline CD4^+^ was the strongest INR predictor, suggesting the value of early ART and individualized care for Indonesian PLHIV.

## 1. Introduction

Immunological non-response (INR) in people living with HIV (PLHIV) undergoing antiretroviral therapy (ART) remains a major clinical challenge, characterized by persistently low CD4^+^ T-cell counts despite sustained viral suppression [1,2]. Globally, INR affects up to 30% of people living with HIV on ART and, in some cohorts, is associated with an approximately three-fold higher risk of mortality, alongside elevated rates of both AIDS- and non-AIDS–related comorbidities [1,3,4]. Mechanistic studies implicate persistent immune activation, microbial translocation, and systemic inflammation as drivers of this phenomenon [5,6].

Despite its global importance, INR remains poorly characterized in low- and middle-income countries (LMICs), where the burden of HIV is greatest [1,7]. Previous studies have associated baseline CD4^+^ count, tuberculosis (TB) co-infection, gender, body mass index (BMI), and socio-economic status with immune recovery [2,8,9,10,11,12,13,14]. However, these predictors have shown inconsistency across settings, reflecting heterogeneity in study design, patient populations, and viral subtype distribution.

Viral genetic diversity, particularly HIV-1 subtypes, has recently emerged as a key determinant of immune recovery. CRF01_AE, the dominant subtype in Southeast Asia and parts of the global epidemic, has been linked to impaired CD4^+^ T-cell restoration and a higher frequency of CXCR4-tropic viruses, which may exacerbate immune dysfunction [15]. Indonesia, which has the third-largest HIV epidemic in Asia and is predominantly affected by CRF01_AE, provides a unique context to investigate the combined influence of demographic, clinical, and viral factors on INR [16,17,18].

A further challenge lies in the absence of standardized INR definitions, which limits comparability across studies. Existing criteria vary widely, using either absolute CD4^+^ thresholds or relative CD4^+^ gains, leading to fragmented evidence and inconsistent risk profiling [19,20].

This study addresses these gaps through a two-decade retrospective cohort analysis of Indonesian PLHIV receiving ART. The novelty lies in two aspects: (i) evaluating INR within a CRF01_AE-predominant population, generating insights with direct relevance to broader global regions; and (ii) being the first study to comprehensively apply and compare four operational INR definitions, thereby enhancing comparability with international research. By integrating demographic, clinical, and virological factors, this work aims to provide a more nuanced understanding of INR predictors and inform strategies to optimize immune recovery in resource-limited and subtype-diverse settings.

## 2. Materials and Methods

### 2.1. Study Design and Population

This study employed a retrospective observational design, utilizing secondary data collected from the medical records of HIV-infected adult patients who initiated antiretroviral therapy (ART) between January 2004 and December 2024 at two major healthcare centers in Surabaya, Indonesia: RSUD Dr. Soetomo and RS Universitas Airlangga (RSUA). After two years of continuous ART, the study population comprised patients who had achieved virological suppression, defined as a plasma HIV-1 RNA viral load of <50 copies/mL. Patients were classified into immunological non-responders (INRs) and immunological responders (IRs) based on four operational definitions of INR, applied after two years of ART among virologically suppressed individuals: (1) INR1 was defined as the failure to increase CD4^+^ T-cell count by ≥100 cells/mm^3^ from baseline within the first two years of ART; (2) INR2 was defined as the failure to reach a total CD4^+^ T-cell count ≥350 cells/mm^3^ within the first two years of ART, regardless of baseline count or CD4^+^ gain; (3) INR3 was defined as meeting either INR1 or INR2; and (4) INR4 was defined as meeting both INR1 and INR2 criteria simultaneously. Patients who did not meet these INR criteria were considered immunological responders. These four operational INR categories were derived from three underlying criteria: CD4^+^ T-cell gain, an absolute CD4^+^ T-cell threshold, and the combination of both, and were used to stratify patients into four INR groups (INR1–INR4). Eligible patients were those aged 18 years or older at the time of ART initiation, with complete baseline and follow-up clinical data, including CD4^+^ T-cell counts and viral load measurements. Exclusion criteria included pregnancy, poor ART adherence (defined as >12 missed doses per month), incomplete medical records, or death within the first two years of ART.

### 2.2. Definitions and Data Collection

The primary outcome was immunological non-response as defined by four operational criteria (INR1–INR4, as detailed above). Independent variables include sex (male vs. female), age (<50 vs. ≥50 years), Mode of HIV Transmission (vaginal sexual transmission, anal sexual transmission, injection drug use, other routes), BMI (underweight vs. non-underweight), Tuberculosis (TB) Status (active TB at ART initiation vs. no active TB at ART initiation), HIV clinical stage (advanced HIV disease (WHO stage 3–4) vs. non-advanced HIV Disease (WHO stage 1–2)), ART regimen (TDF-based regimen vs. non-TDF-based regimen), Educational Attainment (less than a bachelor’s degree vs. bachelor’s degree or higher), and baseline CD4^+^ T-cell count (<200 cells/mm^3^ vs. ≥200 cells/mm^3^). CD4^+^ T-cell counts were measured using flow cytometry (BD FACSLyric™, Becton Dickinson, Franklin Lakes, NJ, USA). Plasma HIV-1 RNA viral load was quantified using the Abbott m2000 RealTime HIV-1 assay (Abbott Molecular Inc., Des Plaines, IL, USA). In addition to the primary analysis of INR predictors, the study examined longitudinal CD4^+^ T-cell recovery between groups as a secondary outcome. CD4^+^ T-cell counts were recorded at ART initiation and regular six-month intervals during the two-year follow-up period. Data were extracted manually from the patients’ electronic and paper-based medical records using a standardized data abstraction form developed for this study. All data were anonymized prior to analysis to maintain patient confidentiality.

### 2.3. Statistical Analysis

Data analysis was conducted using R version 4.4.2, with preliminary data cleaning performed to address missing values and duplicates using dplyr version 1.1.4 and tidyr version 1.3.1. Descriptive statistics were calculated for all variables, with means and standard deviations (SD) for normally distributed numerical data and medians with interquartile ranges for non-normally distributed data. Categorical variables were presented as frequencies and percentages. Bivariate analysis was performed using Mann–Whitney U tests for numerical variables and Chi-square or Fisher’s exact tests for categorical variables. A *p*-value < 0.05 was considered statistically significant. Multivariate logistic regression was conducted for variables with *p* < 0.25 in bivariate analysis to determine independent predictors of INR. Results were expressed as odds ratios (ORs) with 95% confidence intervals (CIs). Missing data were handled using complete-case analysis, wherein only records with complete values for all variables of interest were included in the multivariable models. For the secondary analysis of CD4^+^ T-cell recovery, median CD4^+^ counts were computed at multiple time points (0, 6, 12, 24, 36, and >36 months) for both groups. Trends were visualized, and slope estimates between consecutive intervals were calculated based on group-level medians. No subject-level longitudinal modeling was applied.

## 3. Results

### 3.1. Immunological Response and Non-Response Characteristics Among Indonesian HIV Patients

A total of 464 HIV-positive patients receiving outpatient care were screened for eligibility. Following screening, 82 patients were removed due to incomplete medical records (*n* = 79) or inadequate treatment adherence (*n* = 3). As a result, 382 patients met all inclusion criteria and included into the final analysis. Table 1 presents a comparative overview of clinical and demographic characteristics of patients stratified by immunological response status (IR vs. INR) under four distinct operational definitions (INR1–INR4). In line with the current lack of consensus on a single standard definition of INR, these four INR groups (INR1–INR4) were constructed using three different operational criteria: CD4^+^ T-cell gain, an absolute CD4^+^ T-cell threshold, and their combination, as detailed in the Materials and Methods section. Overall, the proportion of patients classified as immunological non-responders was 13% for INR1 (*n* = 50), 52% for INR2 (*n* = 200), 54% for INR3 (*n* = 207), and 11% for INR4 (*n* = 43), with the remaining patients in each classification fulfilling the criteria for immunological response (IR1–IR4, respectively). There were no missing data for the key variables analyzed, including sex, age, mode of HIV transmission, body mass index (BMI), tuberculosis (TB) status, HIV clinical stage, ART regimen, educational attainment, and baseline CD4^+^ T-cell count. Across all INR definitions, sex distribution, age, and tuberculosis (TB) status did not significantly differ between immunological responders (IRs) and non-responders (INRs). However, several variables demonstrated statistically significant associations with INR outcomes. Older age (≥50 years) was more prevalent among the INR2 and INR3 groups than their respective IR counterparts. The Body Mass Index (BMI) category was significantly associated with INR2 (*p* < 0.001) and INR3 (*p* = 0.002), with underweight patients overrepresented among the associated with INR2 (*p* < 0.001) and INR3 (*p* = 0.002), with underweight patients overrepresented among INR groups. HIV clinical stage was significantly associated with INR2 (*p* < 0.001) and INR3 (*p* < 0.001), with a higher proportion of patients classified as Advanced HIV Disease in INR groups. ART regimen was also associated with INR2 (*p* = 0.031), though not with other definitions. Baseline CD4^+^ count showed consistent and robust associations across INR1 (*p* = 0.008), INR2 (*p* < 0.001), and INR3 (*p* < 0.001), with lower baseline counts significantly more common among INR patients.

### 3.2. Clinical and Demographic Predictors of Immunological Non-Response

Table 2, Table 3, Table 4 and Table 5 present the bivariate and multivariate logistic regression analyses assessing clinical and demographic predictors of immunological non-response (INR), stratified by four operational definitions (INR1–INR4). Baseline CD4^+^ cell count was the most consistent predictor across INR definitions, showing an inverse association with INR1 (Table 2, adjusted OR 0.45, 95% CI: 0.22–0.92, *p* = 0.028) but increased odds of INR for INR2 (Table 3, adjusted OR 5.60, 95% CI: 2.95–10.62, *p* = 0.001) and INR3 (Table 4, adjusted OR 4.46, 95% CI: 2.39–8.29, *p* = 0.001), underscoring its prognostic relevance irrespective of operational definition. Male sex was a significant predictor for INR3 (Table 4, adjusted OR 1.78, 95% CI: 1.08–2.94, *p* = 0.02), but not for INR1, INR2, or INR4. Similarly, underweight BMI demonstrated increased odds of INR in unadjusted models for INR2 and INR3 (crude OR 2.15 and 2.03, respectively; *p* < 0.01). Mode of HIV transmission, specifically anal sexual transmission, was inversely associated with INR2 and INR3. Patients reporting anal sexual transmission had lower odds of INR2 (Table 3, adjusted OR 0.42, 95% CI: 0.19–0.92, *p* = 0.03) and INR3 (Table 4, adjusted OR 0.41, 95% CI: 0.19–0.89, *p* = 0.02), indicating potentially better immunological recovery among this subgroup. Educational attainment was significantly associated with INR2 (Table 3, adjusted OR 2.08, 95% CI: 1.11–3.90, *p* = 0.021) and INR3 (Table 4, adjusted OR 2.62, 95% CI: 1.40–4.91, *p* = 0.003), with higher education paradoxically linked to increased odds of INR. INR4 did not show statistically significant associations with any assessed predictors in adjusted models (Table 5).

### 3.3. Longitudinal CD4^+^ Recovery Patterns in Immunological Responders and Non-Responders

**Figure 1:**
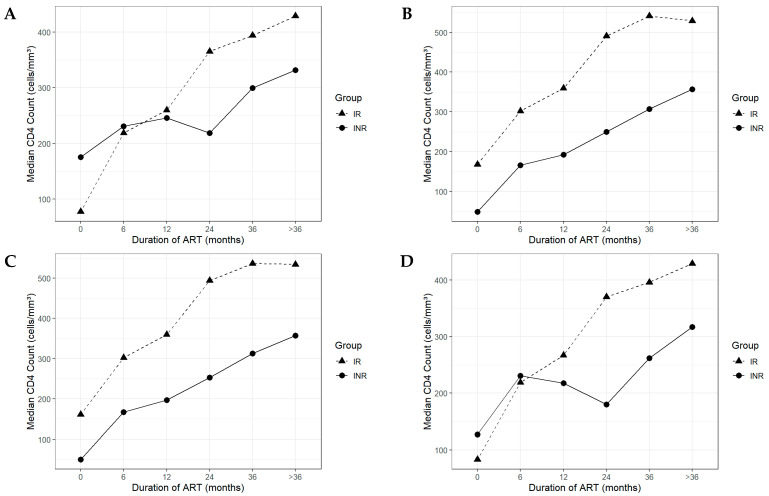
Longitudinal CD4^+^ Recovery Patterns in Immunological Responders (IRs) and Immunological Non-Responders (INRs) Based on Four Definitions. (**A**) INR1 Definition; (**B**) INR2 Definition; (**C**) INR3 Definition; (**D**) INR4 Definition.

**Figure 2:**
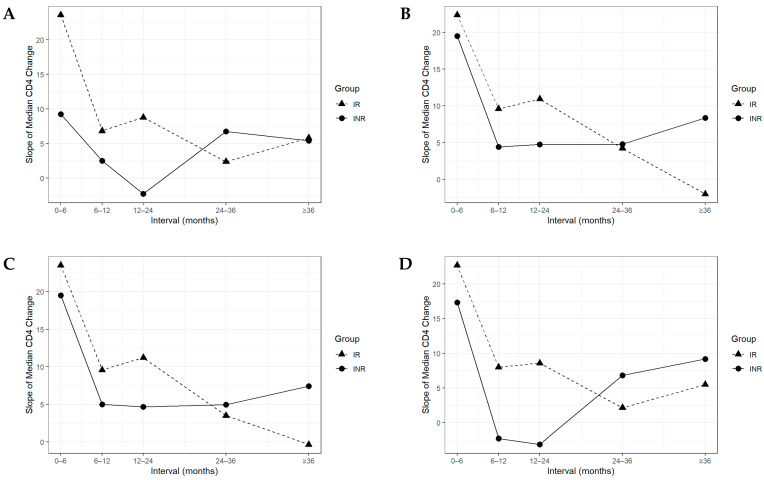
Slope of Median CD4^+^ T-cell Change Across ART Duration Intervals in Immunological Responders (IRs) and Non-Responders (INRs). (**A**) INR1 Definition; (**B**) INR2 Definition; (**C**) INR3 Definition; (**D**) INR4 Definition.

Figure 1 depicts longitudinal CD4^+^ T-cell recovery in immunological responders (IRs) and non-responders (INRs) across four definitions (INR1–INR4). IR consistently achieved superior recovery, although the degree of separation varied by definition. Panel A (INR1) shows comparable baseline counts, but trajectories diverged at month 6: IR gained rapidly between group exhibits a steep incline between 0 and 6 months, while INR progressed slowly, consistent with suboptimal recovery. Panel B (INR2) shows INR initiating ART with lower baseline counts. IR surpassed 350 cells/mm^3^ by month 12, while INR followed a flatter trajectory, failing to reach recovery thresholds despite virological suppression. Panel C (INR3), integrating INR1 and INR2 criteria, shows INR with lower baseline counts and persistent deficits. IR plateaued above 500 cells/mm^3^ by month 36, whereas INR remained below this level throughout follow-up. Panel D (INR4) shows an atypical course, with INR declining between months 6–24 before modest recovery, suggesting a biphasic or unstable trajectory. Figure 2 quantifies these patterns by median CD4^+^ T-cell slopes across ART intervals. The steepest increases occurred in the first 0–6 months, with IR exceeding 20 cells/mm^3^/month and INR ranging 10–20 cells/mm^3^/month. Thereafter, slopes declined in both groups, with IR approaching zero or negative values beyond 36 months (Figure 2B,C).

## 4. Discussion

This study demonstrated that baseline clinical and demographic characteristics significantly influenced immunological recovery in virologically suppressed HIV-infected adults on ART in Indonesia. A low baseline CD4^+^ count (<200 cells/mm^3^) consistently emerged as the strongest predictor of immunological failure across INR definitions. Male sex was also associated with increased odds of poor immune recovery. In contrast, a history of anal sexual contact was associated with a lower risk of INR. Unexpectedly, higher education was linked to increased INR risk. These findings underscore the multifactorial nature of immune recovery and highlight the importance of integrating clinical, demographic, and behavioral factors in assessing ART outcomes. In our cohort, the proportion of patients classified as immunological non-responders ranged from 11% to 54% across INR definitions, with 52% fulfilling the INR2 definition (CD4^+^ ≤350 cells/mm^3^ after ≥2 years of ART), a prevalence that closely mirrors the 53.81% incidence of INR reported by Pang et al. using an equivalent threshold in Guangxi, China [21]. In that study, CRF01_AE was the predominant subtype and was slightly overrepresented among INR compared with IR, whereas several non-CRF01_AE subtypes showed more favorable CD4^+^ T-cell recovery [21]. Although HIV-1 subtype was not directly assessed in our cohort, previous molecular epidemiological studies from Indonesia have consistently shown CRF01_AE to be the dominant circulating subtype [16,17], suggesting that the high INR burden observed here is compatible with immune recovery patterns in a CRF01_AE–dominated epidemic.

Our findings confirm that initiating ART at substantially depleted CD4^+^ levels is strongly associated with poor immunological recovery, consistent with previous studies indicating that late ART initiation frequently results in suboptimal CD4^+^ reconstitution despite virological suppression [22,23,24]. Although thresholds vary across studies, the conclusion remains consistent: individuals initiating ART at lower CD4^+^ counts exhibit impaired immune restoration [25,26,27]. The underlying biological mechanisms are well-described: chronic HIV infection leads to thymic dysfunction and impairs the generation of naïve CD4^+^ T cells, limiting regenerative capacity [28,29]. Compounded by persistent immune activation, microbial translocation, and systemic inflammation, it is often incompletely reversible despite virological control [30,31,32]. In the Indonesian context, delayed HIV diagnosis and ART initiation remain prevalent due to stigma, low testing uptake, and geographic inequities in access [33,34,35], resulting in many patients starting ART at CD4^+^ <200 cells/mm^3^, consistent with local reports of advanced HIV disease [12].

Expanding upon the role of thymic dysfunction in limiting naïve CD4^+^ T-cell regeneration described above, our analysis also identified a higher prevalence of older age (≥50 years) among the INR2 and INR3 groups compared with their corresponding IR counterparts, suggesting that aging-related immunologic changes contribute to incomplete immune reconstitution. Evidence from large clinical cohorts and mechanistic studies indicates that older people living with HIV have attenuated CD4^+^ T-cell recovery after ART initiation compared with younger adults, even when virological suppression is maintained [36,37]. This pattern is consistent with immunosenescence, in which aging is associated with a decline in naïve T-cell production, accumulation of senescent T cells, and reduced capacity to rebuild the CD4^+^ T-cell compartment following immune damage [38,39]. Thymic involution, structural and functional atrophy of the thymus that is further accelerated in chronic HIV infection, leads to diminished thymic output of naïve CD4^+^ T cells and a skewed repertoire dominated by memory phenotypes, thereby limiting immune reconstitution despite effective ART [40,41]. Moreover, older adults are more likely to present with long-standing, advanced HIV disease and severely depressed baseline CD4^+^ counts, which may act synergistically with these age-related changes to increase the likelihood of immunological non-response. Taken together, these mechanisms provide a plausible explanation for the higher proportion of older individuals in our cohort.

Further analysis of longitudinal CD4^+^ recovery patterns revealed clear distinctions between immunological responders (IRs) and non-responders (INRs), as visualized in Figure 1 and Figure 2. While IRs experienced steady and progressive CD4^+^ gains, INRs exhibited stagnant or biphasic trajectories (early rise then plateau/decline). These patterns likely reflect early redistribution followed by insufficient replenishment of memory T-cell subsets [42,43], compounded by persistent systemic inflammation, immunosenescence, and comorbidities [44,45]. Interestingly, baseline CD4^+^ T-cell count <200 cells/mm^3^ was inversely associated with immunological non-response under the INR1 definition, suggesting an apparent protective effect. This paradox is attributable to the relative nature of INR1, which defines non-response as a failure to achieve a ≥100 cells/mm^3^ increase from baseline. Individuals starting ART at very low CD4^+^ levels often demonstrate rapid initial gains due to immune redistribution and expansion of residual naïve and memory T cells, allowing them to meet the 100-cell threshold more readily [10,46]. Conversely, INR1-defined non-responders who exhibit limited early CD4^+^ increases (Figure 1A) and those illustrated in Figure 2A show a sharp decline in the slope of CD4^+^ change after the initial 6 months. Parameters like elevated neutrophil-to-lymphocyte and platelet-to-lymphocyte ratios have been implicated in impaired immune reconstitution, further emphasizing the role of systemic inflammation [47]. Moreover, host genetic factors may also influence recovery trajectories. In Indonesia, polymorphisms in HIV co-receptors such as CCR5 and CXCR4 have been associated with variable immune outcomes in individuals infected with the CRF01_AE subtype [48].

Our analysis also confirms male sex as a significant independent risk factor for immunological non-response. These findings are consistent with previous literature across diverse settings, which has demonstrated inferior immune recovery outcomes among men living with HIV [26,27,49]. Several biological mechanisms may explain the male disadvantage in immune reconstitution. Sex hormones exert critical immunomodulatory effects; estrogen enhances thymic output and supports CD4^+^ T-cell proliferation, while androgens may suppress immune activation pathways [50,51]. Estrogen has also been shown to reinforce mucosal integrity and limit microbial translocation, thereby reducing systemic inflammation, an essential factor in promoting immune restoration during ART [52,53]. Socio-behavioral factors further compound these biological disparities. In Indonesia and similar settings, men are less likely to engage consistently with healthcare services due to cultural norms, stigma, and limited health literacy [54]. Our analysis revealed that male participants were disproportionately represented among individuals with CD4^+^ counts below 200 cells/mm^3^ at ART initiation, a critical determinant of INR. Moreover, men often exhibit poorer adherence, more interruptions, and lower retention [49,55], influenced by psychosocial stressors and stigma [56,57,58,59].

Contrary to prevailing global evidence linking low educational attainment with poor HIV-related health outcomes, our study found that individuals with higher education levels, specifically those holding a bachelor’s degree or above, had an increased risk of immunological non-response (INR). These findings challenge conventional assumptions, as higher education is typically associated with improved health literacy, treatment adherence, and clinical outcomes in people living with HIV [60,61,62,63]. In the Indonesian context, however, higher education may be a proxy for socio-demographic exposures that adversely impact immune recovery. Many university-educated individuals are engaged in high-stress professional environments, particularly in urban areas, where occupational stress may lead to chronic physiological effects, including hormonal dysregulation and systemic inflammation, which impair CD4^+^ T-cell recovery [3,64,65,66]. Moreover, this association persisted even after adjusting for clinical covariates such as baseline CD4^+^ count, sex, and BMI, suggesting the influence of residual or unmeasured psychosocial variables.

In contrast to prevailing assumptions that men who have sex with men (MSM) in LMICs are disproportionately affected by barriers to timely HIV diagnosis and treatment, our study found that a history of homosexual transmission, specifically via anal sexual contact, was significantly associated with a lower risk of immunological non-response (INR). These findings challenge dominant narratives in the literature and highlight the need to account for contextual variations in healthcare access and engagement [67]. In the Indonesian urban setting, MSM may benefit from earlier access to ART through targeted outreach initiatives, peer-led education, and community-based testing supported by non-governmental organizations [68,69]. Greater health literacy and social support within MSM networks are associated with better adherence and outcomes [70,71]. This protective association likely reflects earlier engagement and sustained treatment rather than biological differences, though misclassification and residual confounding remain possible.

This study has several limitations that should be considered when interpreting the findings. First, its retrospective design is inherently susceptible to incomplete or missing data, particularly regarding adherence history, comorbidities, and longitudinal CD4^+^ measurements. Although rigorous data cleaning procedures were applied, the risk of information bias cannot be entirely excluded. Second, over the two-decade study period (2004–2024), Indonesia’s national HIV guidelines and monitoring practices evolved substantially, including shifts in ART regimens and the de-prioritization of routine CD4^+^ testing in favor of viral load monitoring. These policy changes may have introduced inconsistencies in the timing and availability of CD4^+^ data, affecting the assessment of immunologic outcomes. Third, transitions from zidovudine- to tenofovir-based first-line ART regimens may have introduced unmeasured treatment-era effects that were not fully captured in the analyses [16,17]. Fourth, individual-level HIV-1 subtype data were not available for this cohort, and we were therefore unable to directly examine the impact of viral subtype on immunological non-response. However, epidemiological data from Indonesia indicate that HIV-1 CRF01_AE is the predominant circulating subtype [16,17], suggesting that subtype-related biological heterogeneity in this setting is likely to be limited. Despite these limitations, this study has important strengths. A key strength is the use of four clinically relevant definitions of immunological non-response in a well-characterized, multicenter retrospective cohort of virologically suppressed adults, enabling a comprehensive comparative assessment of INR criteria in a resource-limited setting.

## 5. Conclusions

This study identified key predictors of immunological non-response (INR) among virologically suppressed HIV-infected adults in Indonesia. Low baseline CD4^+^ count was the strongest predictor, followed by male sex and higher education, while a history of anal sex was associated with a lower INR risk. These findings underscore the importance of early ART initiation and individualized monitoring, highlighting the value of integrating clinical, demographic, and behavioral factors to optimize ART outcomes in resource-limited settings.

## Figures and Tables

**Table 1:** Clinical and Demographic Characteristics of Patients by Immunological Response Status.

Characteristics	INR1 *n* = 50 (13%)		IR1 *n* = 332 (87%)		*p*-Value	INR2 *n* = 200 (52%)		IR2 *n* = 182 (48%)		*p*-Value	INR3 *n* = 207 (54%)		IR3 *n* = 175 (46%)		*p*-Value	INR4 *n* = 43 (11%)		IR4 *n* = 339 (89%)		*p*-Value
Sex, *n* (%)					0.477					0.161					0.071					0.896
Male	32	(64%)	191	(58%)		124	(62%)	99	(54%)		130	(63%)	93	(53%)		26	(60%)	197	(58%)	
Female	18	(36%)	141	(42%)		76	(38%)	83	(46%)		77	(37%)	82	(47%)		17	(40%)	142	(42%)	
Age, *n* (%)					0.112					0.530					0.379					0.193
≥50 years	6	(12%)	17	(5%)		14	(7%)	9	(5%)		15	(7%)	8	(5%)		5	(12%)	18	(5%)	
<50 years	44	(88%)	315	(95%)		186	(93%)	173	(95%)		192	(93%)	167	(95%)		38	(88%)	321	(95%)	
Age (years), median (IQR)	31	(27, 41.5)	34	(29, 40)	0.32	34	(29, 40)	33	(28, 40)	0.273	34	(29, 40)	33	(28, 39.5)	0.274	31	(27, 41)	34	(29, 40)	0.292
Mode of HIV Transmission, *n* (%)					0.479					**0.048**					0.062					0.268
Vaginal sexual transmission	41	(82%)	269	(81%)		165	(83%)	145	(80%)		171	(83%)	139	(79%)		35	(81%)	275	(81%)	
Anal sexual transmission	3	(6%)	38	(11%)		16	(8%)	25	(14%)		17	(8%)	24	(14%)		2	(5%)	39	(12%)	
Injection drug use	1	(2%)	6	(2%)		2	(1%)	5	(3%)		2	(1%)	5	(3%)		1	(2%)	6	(2%)	
Other routes	5	(10%)	19	(6%)		17	(9%)	7	(4%)		17	(8%)	7	(4%)		5	(12%)	19	(6%)	
Body Mass Index (BMI) Category, *n* (%)					0.77					**<0.001**					**0.002**					0.457
Underweight	17	(34%)	106	(32%)		80	(40%)	43	(24%)		81	(39%)	42	(24%)		16	(37%)	107	(32%)	
Non-underweight	33	(66%)	226	(68%)		120	(60%)	139	(76%)		126	(61%)	133	(76%)		27	(63%)	232	(68%)	
Body Mass Index (kg/m2), median (IQR)	20.2	(17.9, 23.4)	20.3	(17.7, 23.5)	0.905	19.8	(17.6, 21.8)	21.8	(18.6, 24.8)	**<0.001**	19.8	(17.6, 21.8)	21.8	(18.6, 24.8)	**<0.001**	20.1	(17.8, 23.4)	20.3	(17.8, 23.4)	0.935
Tuberculosis (TB) Status, n (%)					0.951					0.749					0.948					1.000
Active TB at ART initiation	5	(10%)	38	(11%)		24	(12%)	19	(10%)		24	(12%)	19	(11%)		5	(12%)	38	(11%)	
No active TB at ART initiation	45	(90%)	294	(89%)		176	(88%)	163	(90%)		183	(88%)	156	(89%)		38	(88%)	301	(89%)	
HIV Clinical Stage, *n* (%)					0.067					**<0.001**					**<0.001**					0.873
Non-Advanced HIV Disease	7	(14%)	22	(7%)		3	(2%)	26	(14%)		7	(3%)	22	(13%)		3	(7%)	26	(8%)	
Advanced HIV Disease	43	(86%)	310	(93%)		197	(99%)	156	(86%)		200	(97%)	153	(87%)		40	(93%)	313	(92%)	
ART Regimen, *n* (%)					0.383					**0.031**					0.05					0.216
TDF-based Regimen	19	(38%)	148	(45%)		77	(38.5%)	90	(49%)		126	(61%)	89	(51%)		15	(35%)	152	(45%)	
Non-TDF-based Regimen	31	(62%)	184	(55%)		123	(61.5%)	92	(51%)		81	(39%)	86	(49%)		28	(65%)	187	(55%)	
Educational Attainment, *n* (%)					0.586					0.081					**0.012**					0.542
Less than a Bachelor’s degree	40	(80%)	276	(83%)		159	(80%)	157	(86%)		162	(78%)	154	(88%)		37	(86%)	279	(82%)	
Bachelor’s degree or higher	10	(20%)	56	(17%)		41	(21%)	25	(14%)		45	(22%)	21	(12%)		6	(14%)	60	(18%)	
Baseline CD4^+^ count, *n* (%)					**0.008**					**<0.001**					**<0.001**					0.360
<200 cells/mm^3^	29	(58%)	254	(77%)		182	(91%)	101	(55%)		182	(88%)	101	(58%)		29	(67%)	254	(75%)	
≥200 cells/mm^3^	21	(42%)	78	(23%)		18	(9%)	81	(45%)		25	(12%)	74	(42%)		14	(33%)	85	(25%)	
Baseline CD4^+^ count (cells/mm^3^), median (IQR)	176	(45.5, 258)	77.5	(24, 190)	**0.005**	49	(19, 121)	168	(53.3, 277)	**<0.001**	49	(19, 129)	168	(50.5, 263)	**<0.001**	127	(43, 226)	83	(26, 198)	0.181

ART, antiretroviral therapy; BMI, Body Mass Index; CD4^+^, cluster of differentiation 4; INR, immunological nonresponder; IR, immunological responder; IQR, interquartile range; *p*-value, probability value; TB, Tuberculosis; TDF, Tenofovir Disoproxil Fumarate.

**Table 2:** Factors associated with poor CD4^+^ cell count recovery based on INR definition 1.

Variable	Crude OR (95% CI)	*p*-Value	Adjusted OR (95% CI)	*p*-Value
Sex		0.388		
Female	Ref.			
Male	1.31 (0.7–2.43)			
Mode of HIV Transmission				
Vaginal sexual transmission	Ref.			
Anal sexual transmission	0.5 (0.15–1.75)	0.29		
Injection drug use	1.09 (0.12–9.31)	0.93		
Other routes	1.72 (0.61–4.87)	0.30		
BMI Category		0.77		
Non-underweight	Ref.			
Underweight	1.09 (0.58–2.06)			
HIV Clinical Stage		0.07		0.64
Non-Advanced HIV Disease	Ref.		Ref.	
Advanced HIV Disease	0.43 (0.17–1.08)		0.78 (0.28–2.2)	
ART Regimen		0.38		
TDF-based Regimen	Ref.			
Non-TDF-based Regimen	1.31 (0.71–2.41)			
Baseline CD4^+^ count		0.006		0.028
>200 cells/mm^3^	Ref.		Ref.	
<200 cells/mm^3^	0.42 (0.22–0.78)		0.45 (0.22–0.92)	
Educational Attainment		0.58		
Less than a Bachelor’s Degree	Ref.			
Bachelor’s Degree or higher	1.23 (0.58–2.6)			

ART, antiretroviral therapy; BMI, Body Mass Index; CD4^+^, cluster of differentiation 4; INR, immunological nonresponder; *p*-value, probability value; Ref., Reference; TDF, Tenofovir Disoproxil Fumarate.

**Table 3:** Factors associated with poor CD4^+^ cell count recovery based on INR definition 2.

Variable	Crude OR (95% CI)	*p*-Value	Adjusted OR (95% CI)	*p*-Value
Sex		0.13		
Female	Ref.		Ref.	0.06
Male	1.36 (0.909–2.06)		1.62 (0.97–2.69)	
Mode of HIV Transmission				
Vaginal sexual transmission	Ref.		Ref.	
Anal sexual transmission	0.56 (0.28–1.09)	0.09	0.42 (0.19–0.92)	0.03
Injection drug use	0.35 (0.06–1.84)	0.21	0.19 (0.03–1.11)	0.06
Other routes	2.13 (0.86–5.29)	0.102	1.07 (0.38–3)	0.89
BMI Category		0.001		0.083
Non-underweight	Ref.		Ref.	
Underweight	2.15 (1.38–3.36)		1.54 (0.94–2.53)	
HIV Clinical Stage		0.001		0.13
Non-Advanced HIV Disease	Ref.		Ref.	
Advanced HIV Disease	10.94 (3.25–36.82)		2.8 (0.72–10.79)	
ART Regimen		0.032		0.13
TDF-based Regimen	Ref.		Ref.	
Non-TDF-based Regimen	1.56 (1.04–2.35)		1.44 (0.89–2.34)	
Baseline CD4^+^ count		0.001		0.001
>200 cells/mm^3^	Ref.		Ref.	
<200 cells/mm^3^	8.1 (4.6–14.27)		5.6 (2.95–10.62)	
Educational Attainment		0.082		0.021
Less than a Bachelor’s Degree	Ref.		Ref.	
Bachelor’s Degree or higher	1.62 (0.94–2.79)		2.08 (1.11–3.9)	

ART, antiretroviral therapy; BMI, Body Mass Index; CD4^+^, cluster of differentiation 4; INR, immunological nonresponder; *p*-value, probability value; Ref., Reference; TDF, Tenofovir Disoproxil Fumarate.

**Table 4:** Factors associated with poor CD4^+^ cell count recovery based on INR definition 3.

Variable	Crude OR (95% CI)	*p*-Value	Adjusted OR (95% CI)	*p*-Value
Sex		0.057		0.02
Female	Ref.		Ref.	
Male	1.48 (0.98–2.24)		1.78 (1.08–2.94)	
Mode of HIV Transmission				
Vaginal sexual transmission	Ref.		Ref.	
Anal sexual transmission	0.57 (0.29–1.11)	0.1	0.41 (0.19–0.89)	0.02
Injection drug use	0.32 (0.06–1.7)	0.18	0.18 (0.03–1.05)	0.05
Other routes	1.97 (0.79–4.9)	0.14	0.96 (0.35–2.69)	0.93
BMI Category		0.002		0.07
Non-underweight	Ref.		Ref.	
Underweight	2.03 (1.3–3.18)		1.56 (0.95–2.54)	
HIV Clinical Stage		0.002		0.65
Non-Advanced HIV Disease	Ref.		Ref.	
Advanced HIV Disease	4.1 (1.71–9.86)		1.26 (0.44–3.59)	
ART Regimen		0.05		0.09
TDF-based Regimen	Ref.		Ref.	
Non-TDF-based Regimen	1.5 (1.00–2.26)		1.49 (0.92–2.39)	
Baseline CD4^+^ count		0.001		0.001
>200 cells/mm^3^	Ref.		Ref.	
<200 cells/mm^3^	5.33 (3.18–8.92)		4.46 (2.39–8.29)	
Educational Attainment				0.003
Less than a Bachelor’s Degree	Ref.		Ref.	
Bachelor’s Degree or higher	2.04 (1.16–3.58)	0.013	2.62 (1.4–4.91)	

ART, antiretroviral therapy; BMI, Body Mass Index; CD4^+^, cluster of differentiation 4; INR, immunological nonresponder; *p*-value, probability value; Ref., Reference; TDF, Tenofovir Disoproxil Fumarate.

**Table 5:** Factors associated with poor CD4^+^ cell count recovery based on INR definition 4.

Variable	Crude OR (95% CI)	*p*-Value	Adjusted OR (95% CI)	*p*-Value
Sex		0.76		
Female	Ref.			
Male	1.1 (0.57–2.1)			
Mode of HIV Transmission				
Vaginal sexual transmission	Ref.		Ref.	
Anal sexual transmission	0.4 (0.09–1.74)	0.22	0.41 (0.09–1.79)	0.23
Injection drug use	1.31 (0.15–11.19)	0.8	1.15 (0.13–10.01)	0.89
Other routes	2.06 (0.72–5.88)	0.17	1.83 (0.62–5.39)	0.26
BMI Category		0.45		
Non-underweight	Ref.			
Underweight	1.28 (0.66–2.48)			
HIV Clinical Stage		0.27		
Non-Advanced HIV Disease	Ref.			
Advanced HIV Disease	0.68 (0.34–1.35)			
ART Regimen		0.218		0.39
TDF-based Regimen	Ref.		Ref.	
Non-TDF-based Regimen	1.51 (0.78–2.94)		1.35 (0.67–2.69)	
Baseline CD4^+^ count		0.293		
>200 cells/mm^3^	Ref.			
<200 cells/mm^3^	0.69 (0.35–1.37)			
Educational Attainment		0.54		
Less than a Bachelor’s Degree	Ref.			
Bachelor’s Degree or higher	0.75 (0.3–1.86)			

ART, antiretroviral therapy; BMI, Body Mass Index; CD4^+^, cluster of differentiation 4; INR, immunological nonresponder; *p*-value, probability value; Ref., Reference; TDF, Tenofovir Disoproxil Fumarate.

## Data Availability

The data presented in this study are available from the corresponding author on reasonable request due to privacy and ethical restrictions.

## Abbreviations

The following abbreviations are used in this manuscript:

AIDS	Acquired Immune Deficiency Syndrome
ART	Antiretroviral therapy
BMI	Body Mass Index
CD4^+^	Cluster of differentiation 4
CI	Confidence interval
HIV	Human Immunodeficiency Virus
INR	Immunological nonresponder
IR	Immunological responder
IQR	Interquartile range
LMICs	Low- and middle-income countries
OR	Odds ratio
PLHIV	People living with HIV
*p*-value	Probability value
Ref.	Reference
RS	Rumah Sakit (hospital)
SD	Standard deviations
TB	Tuberculosis
TDF	Tenofovir Disoproxil Fumarate
WHO	World Health Organization
